# Editorial: Nutrient density: evidence of multisectoral approaches for improved nutrition

**DOI:** 10.3389/fnut.2024.1542624

**Published:** 2024-12-20

**Authors:** Zandile June-Rose Mchiza, Flaminia Ortenzi, Whadi-ah Parker

**Affiliations:** ^1^Non-Communicable Diseases Research Unit, South African Medical Research Council, Cape Town, South Africa; ^2^School of Public Health, University of the Western Cape, Bellville, South Africa; ^3^Global Alliance for Improved Nutrition (GAIN), Geneva, Switzerland; ^4^Amsterdam Public Health Research Institute, Vrije Universiteit Amsterdam, Amsterdam, Netherlands; ^5^Public Health, Societies and Belonging, Human Sciences Research Council, Cape Town, South Africa

**Keywords:** nutrient density, dietary diversity, healthy food access, micronutrients, macronutrients, nutrient adequacy, sustainability, malnutrition

Adequate nutrition is critical throughout the life course, to ensure survival, support growth and development, promote health and wellbeing, and prevent diseases, both infectious and non-communicable (1, 2).

While substantial evidence suggests that the public health burden of micronutrient deficiencies and inadequacies is highest in low- and middle-income countries (LMICs) (3–7), a growing body of literature also highlights the widespread prevalence of micronutrient-related malnutrition (8) in high-income countries (HICs), especially among population groups with increased nutritional requirements, such as infants and young children, adolescent girls, women of reproductive age, pregnant and lactating women, and older people (1, 4, 5, 9–11).

Globalization and urbanization leading to modern lifestyles; insufficient food and nutrition education resulting in distorted perceptions and misinformation; invasive marketing of unhealthy food products; economic constraints; gender dynamics and socio-cultural norms; ethical and environmental concerns; and taste preferences, are among the many factors driving the global nutrition transition toward increasingly processed diets, which are high in energy and low in essential nutrients and fiber (12, 13). Consequently, countries of all income levels are experiencing a growing double burden of malnutrition (14, 15). Moreover, a major emerging challenge is the rapidly increasing influence of multi-national food manufacturing companies, which control significant market shares in both HICs and LMICs and attract consumers of all incomes with highly palatable, affordable, and convenient food products that are nutrient-poor and energy-dense (16).

To counter the negative effects of the global nutrition transition, governments must juggle multiple priorities toward ensuring access to healthy diets for all, while halting the escalating disease burden and minimizing environmental harms associated with current food production and consumption patterns. This complex, multi-dimensional goal was set out in the World Declaration and Plan of Action for Nutrition (17), which highlights the firm commitment of the Food and Agriculture Organization of the United Nations (FAO) and the World Health Organization (WHO) to work together and in close collaboration with Member States “*to ensure sustained nutritional wellbeing for all people, in a peaceful, just and environmentally safe world*.” Improvements in global diet quality would not only result in greater quality of life for billions of people, but also substantial socio-economic (e.g., employment and productivity, healthcare costs) and environmental (e.g., climate change, land and water use, biodiversity) co-benefits (18, 19).

Worthy of acknowledgment is that, changing dietary habits at population and/or household/individual level is extremely challenging, and there is an urgent need for evidence of effective strategies to discourage consumption of unhealthy foods while increasing intake of nutrient-dense options, to curb early mortality and morbidity from malnutrition and diet-related non-communicable diseases (NCDs). In response to this need, public health and nutrition policymakers, researchers, and practitioners at all levels (from global to local) are increasingly engaging with agri-food system actors (e.g., farmers, food manufacturers, distributors, and retailers) to explore options for nutrient-rich, affordable, desirable, convenient, and culture-sensitive foods that can be sustainably produced (20).

Six impactful studies presenting innovative solutions to increase the nutrient density of diets are featured in this Research Topic, a high-level summary of which is provided below.

- The research conducted by Murphy et al. emphasizes the importance of commonly consumed foods as widely available sources of essential nutrients, while also arguing that some of these foods may have negative health implications if not adequately prepared, consumed in excessive quantities, and/or not integrated within a broader healthy diet. For example, the authors suggest beef sandwiches—a US favorite—may positively contribute to macro- and essential micronutrient intake, especially protein, vitamin B12, iron, and choline. However, they also caution US consumers to select low-sodium, lean meat for maximizing nutrient intakes while limiting consumption of saturated fats and sodium, that are associated with NCDs.
- To tackle food and nutrition insecurity through increased consumption of nutrient-dense, affordable, environmentally sustainable, and socially acceptable foods, Drewnowski and Conrad explored the potential of pulses (i.e., beans, lentils, chickpeas, and dried peas) to contribute to alleviating malnutrition associated with accessibility and affordability constraints. Indeed, pulses represent a relatively low-cost, plant-based protein option in many countries worldwide, and contain multiple micronutrients of global health priority. The authors also argue that the production and processing of pulses has a relatively low carbon footprint and requires less energy than other nutrient-rich food groups. Additionally, legumes are a valued culinary ingredient across diverse regions and cultures.
- The study by Bjerknes et al. focused on the role of marine foods in optimizing human nutrient intake. In particular, they emphasized the potential of farmed blue mussels (Mytilus edulis) as an excellent source of essential nutrients commonly lacking in diets globally. Indeed, farmed blue mussels can significantly contribute to meeting daily recommended intakes of essential amino acids and fatty acids (i.e., EPA and DHA), while also serving as sustainable feed for farmed Atlantic salmon.
- Partially aligned with Murphy et al.'s concept, Fatmah and Utomo's research explored the effects on nutritional status of incorporating orange almond potato cookies into the diets of preschool-aged children who are experiencing stunting. The authors conclude that consumption of such cookies can contribute to improvements in nutritional outcomes among the population group considered.
- Several of the six included studies dealt with assessing/qualifying existing methodologies for: (i) quantifying the availability and accessibility of different foods in countries; (ii) preserving the nutrient content of foods during storage; (iii) testing and analyzing the nutrient content of foods; and/or (iv) measuring the environmental impacts and affordability of foods. In particular, Drewnowski and Conrad, Poinsot et al., and Mendoza-Velázquez et al. highlighted the importance of protein-focused nutrient profiling systems for use in food sustainability and affordability assessments, to enable the identification of nutrient-dense, relatively low-cost and sustainable food options. Bjerknes et al., instead, focused on the suitability of different methodologies for preserving the nutritional value of foods during storage, and for testing and analyzing nutrient content (e.g., lipid extraction), especially within the context of animal-source foods such as fish. For instance, they promote the use of steaming and freeze-drying for blue mussels, as these methods effectively preserve mussels' amino acid and fatty acid content during storage. On the contrary, they discourage use of the Folch method for lipid extraction from blue mussels, due to potentially inaccurate and unreliable results.

## Conclusion

This Research Topic, titled “*Nutrient density: evidence of multisectoral approaches for improved nutrition*,” addresses some of the above-mentioned challenges and introduces potential integrated solutions for enhancing nutrient density while being mindful of sustainability priorities. Among others, the following topics are discussed: (i) the persistence of low dietary diversity in both LMICs and HICs; (ii) the potential of commonly consumed foods to positively impact diet quality across countries and regions, but only if consumed in adequate quantities and as part of broader healthy diets; (iii) new and existing methodologies for analyzing the nutrient content of foods and ranking them based on nutritional value and environmental impacts; and (iv) strategies to combat food shortages, improve access to safe and nutritious foods, and promote nutrient adequacy, through sustainable production and supply of healthy foods that minimize environmental harm.
